# Clinical benefit of platinum doublet combination therapy in older adults with advanced non‐small cell lung cancer: A prospective multicenter study by the National Hospital Organization in Japan

**DOI:** 10.1111/1759-7714.14904

**Published:** 2023-04-18

**Authors:** Mototsugu Shimokawa, Masaki Kanazu, Ryusei Saito, Masahide Mori, Atsuhisa Tamura, Yoshio Okano, Yuka Fujita, Takeo Endo, Mitsuru Motegi, Shohei Takata, Toshiyuki Kita, Noriaki Sukoh, Fumitaka Mizuki, Mitsuhiro Takenoyama, Shinji Atagi

**Affiliations:** ^1^ Department of Biostatistics Yamaguchi University Graduate School of Medicine Yamaguchi Japan; ^2^ Clinical Research Institute National Hospital Organization Kyushu Cancer Center Fukuoka Japan; ^3^ Department of Thoracic Oncology National Hospital Organization Osaka Toneyama Medical Center Toyonaka Japan; ^4^ Division of Respiratory Medicine National Hospital Organization Shibukawa Medical Center Shibukawa Japan; ^5^ Department of Respiratory Diseases National Hospital Organization Tokyo National Hospital Tokyo Japan; ^6^ Division of Pulmonary Medicine National Hospital Organization Kochi Hospital Kochi Japan; ^7^ Department of Respiratory Medicine National Hospital Organization Asahikawa Medical Center Asahikawa Japan; ^8^ Department of Respiratory Medicine National Hospital Organization Mito Medical Center Ibaraki Japan; ^9^ Department of Respiratory Medicine National Hospital Organization Takasaki General Medical Center Takasaki Japan; ^10^ Department of Respiratory Medicine National Hospital Organization Fukuokahigashi Medical Center Fukuoka Japan; ^11^ Department of Respiratory Medicine National Hospital Organization Kanazawa Medical Center Kanazawa Japan; ^12^ Department of Respiratory Medicine National Hospital Organization Hokkaido Medical Center Sapporo Japan; ^13^ Center for Clinical Research Yamaguchi University Hospital Yamaguchi Japan; ^14^ Department of Thoracic Oncology National Hospital Organization Kyushu Cancer Center Fukuoka Japan; ^15^ Department of Thoracic Oncology National Hospital Organization Kinki‐Chuo Chest Medical Center Sakai Japan

**Keywords:** non‐small cell lung cancer, older patients, platinum doublet therapy, single‐agent chemotherapy

## Abstract

**Background:**

Previous trials suggest that older adults with non‐small cell lung cancer (NSCLC) derive benefit from platinum doublet combination therapy, but its superiority is controversial. Although geriatric assessment variables are used to assess the individual risk of severe toxicity and clinical outcomes in older patients, the standard first‐line treatment is still debated. Therefore, we aimed to identify the risk factors for clinical outcomes in older patients with NSCLC.

**Methods:**

Patients aged ≥75 years with advanced NSCLC treated at any of 24 National Hospital Organization institutions completed a pre‐first‐line chemotherapy assessment, including patient characteristics, treatment variables, laboratory test values, and geriatric assessment variables. We evaluated whether these variables were the risk factors for progression‐free survival (PFS) and overall survival (OS).

**Results:**

A total of 148 patients with advanced NSCLC were treated with combination therapy (*n* = 90) or monotherapy (*n* = 58). Median PFS was 5.3 months and OS was 13.6 months. We identified that hypoalbuminemia (hazard ratio [HR] 2.570, 95% confidence interval [CI]: 1.117–5.913, *p* = 0.0264) was a risk factor for PFS and monotherapy (HR 1.590, 95% CI: 1.070–2.361, *p* = 0.0217), lactate dehydrogenase (HR 3.682, 95% CI: 1.013–13.39, *p* = 0.0478), and high C‐reactive protein (HR 2.038, 95% CI: 1.141–3.642, *p* = 0.0161) were risk factors for OS. The median OS was significantly longer in patients treated with combination therapy than in those who received monotherapy (16.5 months vs. 10.3 months; HR 0.684, 95% CI: 0.470–0.995, *p* = 0.0453).

**Discussion:**

Platinum doublet combination therapy may be beneficial in older patients with NSCLC. Identification of risk factors will assist in the development of a personalized treatment strategy.

## Graphical abstract

Here, we report that platinum doublet combination therapy may be beneficial in older patients with NSCLC. The OS was significantly longer in patients treated with platinum doublet combination therapy than in those who received monotherapy. Moreover, we identified several laboratory values that reflect prolonged exhaustion owing to inflammation and might be predictors of outcomes in older patients with NSCLC.

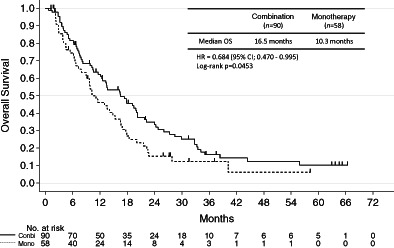

## INTRODUCTION

Lung cancer is the leading cause of cancer‐related deaths worldwide, and the majority of patients diagnosed with the disease have non‐small cell lung cancer (NSCLC).^1^ About 70% of patients with NSCLC are diagnosed at an advanced stage, and the median age at diagnosis is 70 years.^2^ Although systemic chemotherapy is one of the therapeutic options available for patients with advanced NSCLC, the standard first‐line treatment for older patients is still debated. Previous trials have suggested that older patients with NSCLC benefit from platinum doublet combination therapy, whereas its superiority continues to be debated.^3^, ^4^, ^5^, ^6^ An explanation for this controversy is that there is considerable heterogeneity in the physiological changes that occur with aging. Furthermore, a low number of “fit” older patients are enrolled in clinical trials. Therefore, it is difficult to predict the tolerability of chemotherapy in “unfit” older adult patients in clinical practice, because they are more vulnerable to chemotherapy‐related adverse events than “fit” older adult patients.

Age is an important factor in management decisions because of the complex interplay between normal age‐related decline and comorbidities. The Karnofsky performance status or Eastern Cooperative Oncology Group performance status (ECOG‐PS) is used in patients to predict treatment toxicity and survival.^7^, ^8^ However, these tools were validated in younger adults and are not suitable for predicting vulnerability to chemotherapy in older patients. Other factors, including comorbidity, nutrition, physical and cognitive function, and social support, also correlate with toxicity of therapy and cancer outcomes.^9^ The Comprehensive Geriatric Assessment (CGA), which is a compilation of standardized tools for assessment of these factors, can help to predict mortality in older patients with cancer.^10^, ^11^, ^12^, ^13^

Although the CGA is too complicated for use in daily clinical practice, it has been validated among oncologists.^14^, ^15^ Furthermore, several studies have investigated how to predict the risk of chemotherapy toxicity and found that a certain subgroup of older patients are more vulnerable to adverse events from chemotherapy.^16^, ^17^, ^18^ We have previously reported a risk stratification system for prediction of vulnerability to chemotherapy in older patients with NSCLC.^19^ In this study, we evaluated whether several variables, including patients' characteristics and the treatment variables, were the risk factors for progression‐free survival (PFS) and overall survival (OS).

## METHODS

### Patients

In total, 354 patients from any of 24 National Hospital Organization institutions were enrolled in this prospective study between April 2013 and March 2017. A total of 148 of these patients were aged ≥75 years and had histologically or cytologically proven advanced NSCLC (according to the TNM classification, seventh edition) and were treated with cytotoxic chemotherapy (platinum doublet therapy or a single agent) as first‐line therapy. Patients were excluded from the study if they had had active malignancy within the previous 5 years, a history of chemotherapy, had massive pleural or pericardial effusion or ascites, or had received radiation therapy to the lung. The study was approved by the National Hospital Organization Central Review Board and conducted in accordance with the Declaration of Helsinki and ethical guidelines for clinical research (UMIN000010384). All patients provided their written informed consent before enrollment.

### Study schema

All patients completed a pre‐first‐line chemotherapy assessment, which included the characteristics of the cancer (tumor type, stage, and driver mutation status), treatment variables, laboratory test values, and geriatric assessment variables. The ability to perform activities of daily living was assessed using the Barthel Index.^20^ Independence in everyday living and dementia were evaluated by physicians. Hearing and falls in the previous 6 months were evaluated by self‐report or by the family. The patients were followed through one cycle of chemotherapy to monitor for grade 3 (severe) to grade 5 (death) adverse events according to the National Cancer Institute Common Terminology Criteria for Adverse Events, version 4.0. The antitumor response to treatment was assessed on the basis of the Response Evaluation Criteria in Solid Tumors (version 1.1) using computed tomography scans. PFS was defined as the interval between treatment and the date of the first documented tumor progression, as determined by the attending physicians, or death from any cause, whichever occurred first. For cases without computed tomography examination but wherein clinical symptoms or findings on a chest radiograph suggested progression of disease, progression disease onset was defined as the date when the physician clinically evaluated the progression of disease. OS was defined as the interval between the date of diagnosis and date of death or date of last follow‐up for censored patients.

### Statistical analysis

Patient characteristics are summarized using descriptive statistics or contingency tables. Associations between treatments and patient characteristics were examined using the unpaired *t*‐test for continuous variables and the chi‐squared test for categorical variables. PFS and OS were estimated using the Kaplan–Meier method, and the survival curves were compared with the log‐rank test and a Cox proportional hazards model. The risk factors for PFS and OS were evaluated using a Cox proportional hazards model. In the multivariable analysis, all variables were evaluated for univariate analysis were selected. All statistical analyses were performed using SAS 9.4 (SAS Institute Inc.). A two‐sided *p*‐value of ≤ 0.05 was considered statistically significant.

## RESULTS

### Patient and treatment characteristics

A total of 148 patients were included in the analysis (Table 1). The median age was 78 years (range, 75–88). The proportion of patients with stage IIIB NSCLC was 12.8%, and those of patients including stage IV and recurrence was 87.2%. About four‐fifths of the patients were male (81.8%), and the most common tumor type was adenocarcinoma (68.2%). In terms of first‐line cytotoxic chemotherapy, more patients received platinum doublet combination chemotherapy (60.8%) than monotherapy (39.2%). About half of the patients (48.0%) were treated with standard doses for nonelderly patients.

**TABLE 1:** Patient demographics and clinical characteristics at baseline.

Characteristics	Monotherapy (*N* = 58)	Combination (*N* = 90)	Overall (*N* = 148)	*p*‐value
Age				
Mean ± SD	80.1 ± 3.2	77.7 ± 2.4	78.6 ± 3.0	<0.0001
Median (range)	80.0 (75.0–88.0)	77.0 (75.0–86.0)	78 (75.0–88.0)	
Sex, *n* (%)				0.8551
Male	47 (81.0)	74 (82.2)	121 (81.8)	
Female	11 (19.0)	16 (17.8)	27 (18.2)	
Stage, *n* (%)				0.0699
III B	3 (5.2)	16 (17.8)	19 (12.8)	
IV	48 (82.8)	62 (68.9)	110 (74.3)	
recurrence	7 (12.1)	12 (13.3)	19 (12.8)	
Histology, *n* (%)				0.9307
Adenocarcinoma	39 (67.2)	62 (68.9)	101 (68.2)	
Non‐small cell carcinoma	4 (6.9)	7 (7.8)	11 (7.4)	
Squamous cell carcinoma	15 (25.9)	21 (23.3)	36 (24.3)	
Mutation status (EGFR or ALK), *n* (%)				0.8742
Mutation	4 (6.9)	8 (8.9)	12 (8.1)	
Wild‐type	29 (50.0)	42 (46.7)	71 (48.0)	
Unknown	25 (43.1)	40 (44.4)	65 (43.9)	
BMI, *n* (%)				0.2054
<22	30 (51.7)	37 (41.1)	67 (45.3)	
≥22	28 (48.3)	53 (58.9)	81 (54.7)	
ECOG‐PS, *n* (%)				0.4683
0–1	53 (91.4)	85 (94.4)	138 (93.2)	
≥2	5 (8.6)	5 (5.6)	10 (6.8)	
CCI, *n* (%)				0.3559
0–1	47 (81.0)	78 (86.7)	125 (84.5)	
≥2	11 (19.0)	12 (13.3)	23 (15.5)	
Bodyweight loss, *n* (%)				0.9950
<5%	49 (84.5)	76 (84.4)	125 (84.5)	
≥5%	9 (15.5)	14 (15.6)	23 (15.5)	
Frail, *n* (%)				0.3559
1	47 (81.0)	78 (86.7)	125 (84.5)	
≥2	11 (19.0)	12 (13.3)	23 (15.5)	
Recognition, *n* (%)				0.1649
1	53 (91.4)	87 (96.7)	140 (94.6)	
≥2	5 (8.6)	3 (3.3)	8 (5.4)	
Barthel Index, *n* (%)				0.6647
<85	4 (6.9)	8 (8.9)	12 (8.1)	
≥85	54 (93.1)	82 (91.1)	136 (91.9)	
MMSE, *n* (%)				0.8265
<27	21 (36.2)	31 (34.4)	52 (35.1)	
≥27	37 (63.8)	59 (65.6)	96 (64.9)	
Dose reduction, *n* (%)				
No	47 (81.0)	24 (26.7)	71 (48.0)	<0.0001
Yes	11 (19.0)	66 (73.3)	77 (52.0)	
Anemia, *n* (%)				0.9198
0–1	55 (94.8)	85 (94.4)	140 (94.6)	
≥2	3 (5.2)	5 (5.6)	8 (5.4)	
Hypoalbuminemia, *n* (%)				0.7803
0–1	50 (86.2)	79 (87.8)	129 (87.2)	
≥2	8 (13.8)	11 (12.2)	19 (12.8)	
Creatinine, *n* (%)				0.9778
None	51 (87.9)	79 (87.8)	130 (87.8)	
≥1	7 (12.1)	11 (12.2)	18 (12.2)	
LDH, *n* (%)				0.6534
<460	56 (96.6)	88 (97.8)	144 (97.3)	
≥460	2 (3.4)	2 (2.2)	4 (2.7)	
CRP, *n* (%)				0.8509
<3	45 (77.6)	71 (78.9)	116 (78.4)	
≥3	13 (22.4)	19 (21.1)	32 (21.6)	

Abbreviations: BMI, body mass index; CCI, Charlson Comorbidity Index; CRP, C‐reactive protein; ECOG‐PS, Eastern Cooperative Oncology Group performance status; LDH, lactate dehydrogenase; MMSE, Mini‐Mental State Examination; SD, standard deviation.

### Geriatric assessment

A total of 23 patients (15.5%) were at high risk of complications (Charlson Comorbidity Index^21^ ≥2 points). Body mass index (BMI, calculated as kg/m^2^) ranged from 15.5 to 32.9, with about half (54.7%) of the patients having a BMI ≥22. Twenty‐three patients (15.5%) had experienced weight loss of ≥5% within the previous 6 months. Although most patients had good performance status (ECOG‐PS 0/1, 93.2%), patients with poor performance status (ECOG‐PS ≥2, 6.8%) were also enrolled. Twenty‐three patients (15.5%) were limited in their ability to perform activities of daily living. Twelve patients (8.1%) had a Barthel Index score of <85 points. About one‐third of the patients (35.1%) scored <27 points on the Mini‐Mental State Examination.^22^ Eight patients (5.4%) had trouble in everyday living because of cognitive dysfunction.

### Effects of combination therapy and monotherapy on PFS and OS

**FIGURE 1:**
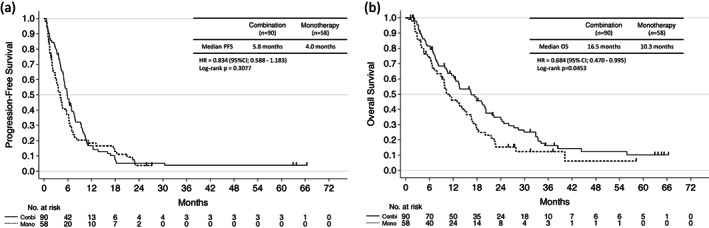
Progression‐free survival and overall survival in the single‐agent chemotherapy group and the platinum doublet combination therapy group. CI, confidence interval; HR, hazard ratio; OS, overall survival; PFS, progression‐free survival.

The median PFS in patients treated with platinum doublet combination therapy was 5.8 months (95% CI: 4.9–7.0), which was not significantly different from the median PFS of 4.0 months (95% CI: 2.8–5.7) in patients who received monotherapy (HR 0.834, 95% CI: 0.588–1.183, *p* = 0.3077) (Figure 1a). However, the median OS in patients treated with combination therapy was 16.5 months (95% CI: 12.1–20.2), which was significantly longer than that of 10.3 months (95% CI: 7.9–15.4) in patients who received monotherapy (HR 0.684, 95% CI: 0.470–0.995, *p* = 0.0453) (Figure 1b).

The overall response rate was higher in the combination cohort than in the single‐agent cohort (31.1% vs. 13.8%). The disease control rates were similar between these cohorts (Table 2).

**TABLE 2:** Response rates in the single‐agent chemotherapy group and the platinum doublet combination therapy group.

Response rate	Monotherapy (*N* = 58), *n* (%)	Combination (*N* = 90), *n* (%)	Overall (*N* = 148), *n* (%)
CR	1 (1.7)	1 (1.1)	2 (1.4)
PR	7 (12.1)	27 (30.0)	34 (23.0)
SD	34 (58.6)	42 (46.7)	76 (51.4)
PD	14 (24.1)	19 (21.1)	33 (22.3)
NE	2 (3.4)	1 (1.1)	3 (2.0)
Overall response rate			
*n* (%)	8 (13.8)	28 (31.1)	36 (24.4)
[95% CI]	[6.1–25.4]	[21.8–41.7]	[17.7–32.1]
Disease control rate			
*n* (%)	42 (72.4)	70 (77.8)	112 (75.7)
[95% CI]	[59.1–83.3]	[67.8–85.9]	[68.0–82.4]

Abbreviations: CI, confidence interval; CR, complete response; NE, not evaluable; PD, progressive disease; PR, partial response; SD, stable disease.

### Toxicity of chemotherapy

A total of 62 patients developed severe hematological toxicity (grade 3–5 according to the National Cancer Institute Common Terminology Criteria for Adverse Events, version 4.0). The frequency of severe hematological toxicity was higher in patients treated with single‐agent chemotherapy than in those treated with platinum doublet combination therapy (51.7% vs. 35.6%). In terms of nonhematological toxicity, adverse events emerged in most cases at any grades. The frequency of severe nonhematological toxicity was not high and was similar between the combination and monotherapy groups (Table 3).

**TABLE 3:** Adverse events.

Adverse event	Monotherapy (*N* = 58)	Combination (*N* = 90)	Overall (*N* = 148)
Hematological toxicity, *n* (%)			
Any	49 (84.5)	83 (92.2)	132 (89.2)
≥grade 3	30 (51.7)	32 (35.6)	62 (41.9)
Non‐hematological toxicity, *n* (%)			
Any	55 (94.8)	87 (96.7)	142 (95.9)
≥grade 3	8 (13.8)	13 (14.4)	21 (14.2)

### Risk factors for PFS and OS

**FIGURE 2:**
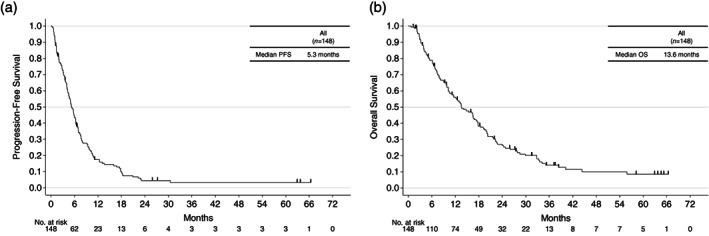
Progression‐free survival and overall survival in the entire study population. OS, overall survival; PFS, progression‐free survival.

The median follow‐up duration was 11.9 months. Median PFS was 5.3 months (95% CI: 4.4–6.3) and median OS was 13.6 months (95% CI: 10.5–17.3) for all patients (Figure 2). Patient sex, Mini‐Mental State Examination score, performance status, Charlson Comorbidity Index value, weight loss, frailty, recognition, Barthel Index, MMSE, anemia, hypoalbuminemia, creatinine, lactate dehydrogenase (LDH), C‐reactive protein (CRP), and platinum doublet combination therapy or single‐agent chemotherapy were evaluated as potential risk factors in the Cox proportional hazards model. Hypoalbuminemia was the only independent risk factor for PFS (hazard ratio [HR] 2.570, 95% CI: 1.117–5.913, *p* = 0.0264) (Table 4). Independent risk factors for OS were monotherapy (HR 1.590, 95% CI: 1.070–2.361, *p* = 0.0217), high LDH (HR 3.682, 95% CI: 1.013–13.39, *p* = 0.0478), and high CRP (HR 2.038, 95% CI: 1.141–3.642, *p* = 0.0161) (Table 4).

**TABLE 4:** Risk factors for progression‐free survival and overall survival.

	Progression‐free survival				Overall survival			
	Univariate		Multivariate		Univariate		Multivariate	
Risk factor	OR (95% CI)	*p*‐value	OR (95% CI)	*p*‐value	OR (95% CI)	*p*‐value	OR (95% CI)	*p*‐value
Sex: male vs. female	1.164 (0.735–1.843)	0.5176	1.174 (0.712–1.935)	0.5289	1.296 (0.792–2.121)	0.3023	1.403 (0.799–2.463)	0.2392
BMI: ≥22 vs. <22	1.130 (0.804–1.587)	0.4823	0.969 (0.662–1.419)	0.8733	1.237 (0.857–1.786)	0.2559	0.991 (0.650–1.511)	0.9660
ECOG‐PS: ≥2 vs. 0–1	3.474 (1.719–7.022)	0.0005	2.619 (0.854–8.032)	0.0921	3.145 (1.520–6.507)	0.0020	2.353 (0.670–8.257)	0.1817
CCI: 0–1 vs. ≥2	1.017 (0.638–1.620)	0.9441	0.807 (0.464–1.404)	0.4477	1.217 (0.717–2.065)	0.4662	0.936 (0.510–1.717)	0.8306
Bodyweight loss: ≥5% vs. <5%	1.108 (0.681–1.803)	0.6793	0.752 (0.405–1.394)	0.3652	1.508 (0.899–2.532)	0.1197	1.155 (0.606–2.198)	0.6618
Frail: ≥2 vs. 1	1.239 (0.770–1.995)	0.3771	1.667 (0.741–3.752)	0.2166	1.519 (0.904–2.550)	0.1143	1.376 (0.569–3.325)	0.4787
Recognition 1 vs. ≥2	1.934 (0.851–4.395)	0.1153	2.594 (0.895–7.516)	0.0791	1.173 (0.478–2.883)	0.7275	1.209 (0.394–3.712)	0.7397
Barthel Index: <85 vs. ≥85	1.369 (0.738–2.542)	0.3195	0.908 (0.371–2.221)	0.8333	1.668 (0.893–3.114)	0.1084	0.861 (0.326–2.276)	0.7634
MMSE: ≥27 vs. <27	1.184 (0.829–1.692)	0.3533	1.063 (0.694–1.627)	0.7803	1.110 (0.758–1.626)	0.5920	1.079 (0.683–1.705)	0.7439
Anemia: ≥2 vs. 0–1	3.014 (1.457–6.231)	0.0029	0.999 (0.374–2.666)	0.9977	4.742 (2.008–11.19)	0.0004	2.745 (0.992–7.598)	0.0519
Hypoalbuminemia: ≥2 vs. 0–1	3.452 (2.029–5.876)	<0.0001	2.570 (1.117–5.913)	0.0264	2.882 (1.647–5.042)	0.0002	1.309 (0.567–3.021)	0.5279
Creatinine: None vs. ≥1	1.304 (0.742–2.291)	0.3563	1.433 (0.748–2.744)	0.2778	1.480 (0.790–2.772)	0.2208	2.158 (0.980–4.752)	0.0563
LDH: ≥460 vs. <460	2.195 (0.691–6.979)	0.1826	2.751 (0.807–9.384)	0.1059	1.868 (0.591–5.903)	0.2874	3.682 (1.013–13.39)	0.0478
CRP: ≥3 vs. <3	2.547 (1.661–3.905)	<0.0001	1.612 (0.902–2.881)	0.1068	2.525 (1.620–3.935)	<0.0001	2.038 (1.141–3.642)	0.0161
Single agent chemotherapy vs. platinum doublet combination therapy	0.834 (0.588–1.183)	0.3098	0.836 (0.572–1.222)	0.3560	0.684 (0.470–0.995)	0.0468	0.629 (0.423–0.934)	0.0217

Abbreviations: BMI, body mass index; CCI, Charlson Comorbidity Index; CI, confidence interval; CRP, C‐reactive protein; ECOG‐PS, Eastern Cooperative Oncology Group performance status; LDH, lactate dehydrogenase; MMSE, Mini‐Mental State Examination.

## DISCUSSION

The correlation between CGA and clinical outcomes in older patients with cancer has previously been investigated.^9^, ^10^, ^11^, ^12^, ^13^ There is considerable heterogeneity in older patients with cancer in terms of physiological changes, and it is difficult to identify factors that predict clinical outcomes, including the negative effects of adverse events. Previous studies have identified several risk factors that could predict the frequency of severe adverse events in older patients.^16^, ^17^, ^18^ Furthermore, we have developed a risk stratification tool to predict vulnerability to chemotherapy in older patients with NSCLC.^19^ However, long‐term survival is the essential factor when considering the treatment strategy.

Two recent randomized Phase III trials that compared docetaxel with platinum doublet combination chemotherapy (carboplatin plus pemetrexed, and carboplatin plus nab‐paclitaxel) found that combination chemotherapy was tolerable and highly effective for “fit” older adults.^4^, ^5^ A certain number of “unfit” older patients were included in our study, and our findings suggested the efficacy of combination chemotherapy in the clinical setting, especially in terms of survival. Although the difference may be more pronounced in the long‐term survival than PFS, further discussion is limited as there is a bias in the first‐line therapy regimen, which is chosen by the attending physicians. Furthermore, the Japanese population appears to be more susceptible to toxicities from docetaxel.^23^ Docetaxel or docetaxel plus bevacizumab was administered in about half of the patients treated with monotherapy in our study (Table S1). This high number of patients in the docetaxel group may be the reason why monotherapy emerged as one of the risk factors for overall survival.

In this study, we also identified high LDH and CRP levels as risk factors for OS. However, LDH is elevated not only in patients with cancer but also in those with other diseases, so the prognostic role of LDH in patients with lung cancer is not conclusive.^24^, ^25^, ^26^ In addition to survival, LDH is one of the factors predicting the risk of chemotherapy toxicity in older patients with cancer.^16^ The CRP to albumin ratio (high CRP and hypoalbuminemia), reflecting prolonged exhaustion owing to inflammation, may be a potential prognostic factor in patients with cancer.^27^, ^28^, ^29^ In addition to CRP, hypoalbuminemia was extracted as an independent risk factor for PFS in our study. Nutritional status as well as tumor inflammation may be more relevant to clinical outcome.

This study had several limitations. First, there was a degree of bias in the treatment variables because the decision regarding selection of the first‐line chemotherapy regimen or dose de‐escalation was made by the physicians, as in clinical practice. Second, patients with NSCLC treated with immune checkpoint inhibitors (ICIs) as first‐line therapy were not included. As previously reported, pembrolizumab has a clinical benefit in patients with advanced NSCLC, regardless of patient age.^30^, ^31^ Although the combination of platinum doublet chemotherapy and an ICI has emerged as one of the standard therapies for patients with advanced NSCLC,^32^, ^33^, ^34^, ^35^ its safety in older patients is uncertain. When choosing a therapeutic strategy involving an ICI, we should understand the factors influencing clinical outcomes before starting chemotherapy.

In conclusion, our study has identified several laboratory values that reflect prolonged exhaustion owing to inflammation and might be predictors of outcomes in older patients with NSCLC. Moreover, platinum doublet combination therapy may be of benefit in this population in the clinical setting.

## AUTHOR CONTRIBUTIONS

Conceptualization: Mototsugu Shimokawa, Masaki Kanazu, Shinji Atagi. Data curation: Ryusei Saito, Masahide Mori, Atsuhisa Tamura, Yoshio Okano, Yuka Fujita, Takeo Endo, Mitsuru Motegi, Shohei Takata, Toshiyuki Kita, Noriaki Sukoh, Mitsuhiro Takenoyama, Shinji Atagi. Formal analysis: Mototsugu Shimokawa, Masaki Kanazu, Fumitaka Mizuki. Funding acquisition: Masaki Kanazu. Investigation: Mototsugu Shimokawa, Masaki Kanazu. Methodology: Mototsugu Shimokawa, Masaki Kanazu, Shinji Atagi. Supervision: Fumitaka Mizuki, Shinji Atagi. Validation: Fumitaka Mizuki, Mototsugu Shimokawa. Visualization: Mototsugu Shimokawa, Masaki Kanazu. Writing: Mototsugu Shimokawa, Masaki Kanazu. Review and editing: Ryusei Saito, Masahide Mori, Atsuhisa Tamura, Yoshio Okano, Yuka Fujita, Takeo Endo, Mitsuru Motegi, Shohei Takata, Toshiyuki Kita, Noriaki Sukoh, Fumitaka Mizuki, Mitsuhiro Takenoyama, Shinji Atagi.

## FUNDING INFORMATION

This study was supported financially by the National Hospital Organization in Japan.

## CONFLICT OF INTEREST STATEMENT

The authors declare that they have no known competing financial interests or personal relationships that could have appeared to influence the work reported in this paper.

## Supporting information


**Table S1.** Detail of regimen
