# Correction to “Clinical benefit of platinum doublet combination therapy in older adults with advanced non‐small cell lung cancer: A prospective multicenter study by the National Hospital Organization in Japan”

**DOI:** 10.1111/1759-7714.15047

**Published:** 2023-07-24

Shimokawa M, Kanazu M, Saito R, Mori M, Tamura A, Okano Y, et al. Clinical benefit of platinum doublet combination therapy in older adults with advanced non‐small cell lung cancer: A prospective multicenter study by the National Hospital Organization in Japan. Thorac Cancer. 2023;14(17):1597–605. https://doi.org/10.1111/1759-7714.14904

In Table 4, all instances of “OR (95% CI)” in the table heading has been corrected to “HR (95% CI)”. The revised table is shown below:

TABLE 4. Risk factors for progression‐free survival and overall survival.

**Table:** 

	Progression‐free survival				Overall survival			
	Univariate		Multivariate		Univariate		Multivariate	
	HR (95% CI)	*p*‐value	HR (95% CI)	*p*‐value	HR (95% CI)	*p*‐value	HR (95% CI)	*p*‐value
Sex: male vs. female	1.164 (0.735–1.843)	0.5176	1.174 (0.712–1.935)	0.5289	1.296 (0.792–2.121)	0.3023	1.403 (0.799–2.463)	0.2392
BMI: ≥22 vs. <22	1.130 (0.804–1.587)	0.4823	0.969 (0.662–1.419)	0.8733	1.237 (0.857–1.786)	0.2559	0.991 (0.650–1.511)	0.9660
ECOG‐PS: ≥2 vs. 0–1	3.474 (1.719–7.022)	0.0005	2.619 (0.854–8.032)	0.0921	3.145 (1.520–6.507)	0.0020	2.353 (0.670–8.257)	0.1817
CCI: 0–1 vs. ≥2	1.017 (0.638–1.620)	0.9441	0.807 (0.464–1.404)	0.4477	1.217 (0.717–2.065)	0.4662	0.936 (0.510–1.717)	0.8306
Bodyweight loss: ≥5% vs. <5%	1.108 (0.681–1.803)	0.6793	0.752 (0.405–1.394)	0.3652	1.508 (0.899–2.532)	0.1197	1.155 (0.606–2.198)	0.6618
Frail: ≥2 vs. 1	1.239 (0.770–1.995)	0.3771	1.667 (0.741–3.752)	0.2166	1.519 (0.904–2.550)	0.1143	1.376 (0.569–3.325)	0.4787
Recognition: 1 vs. ≥2	1.934 (0.851–4.395)	0.1153	2.594 (0.895–7.516)	0.0791	1.173 (0.478–2.883)	0.7275	1.209 (0.394–3.712)	0.7397
Barthel Index: <85 vs. ≥85	1.369 (0.738–2.542)	0.3195	0.908 (0.371–2.221)	0.8333	1.668 (0.893–3.114)	0.1084	0.861 (0.326–2.276)	0.7634
MMSE: ≥27 vs. <27	1.184 (0.829–1.692)	0.3533	1.063 (0.694–1.627)	0.7803	1.110 (0.758–1.626)	0.5920	1.079 (0.683–1.705)	0.7439
Anemia: ≥2 vs. 0–1	3.014 (1.457–6.231)	0.0029	0.999 (0.374–2.666)	0.9977	4.742 (2.008–11.19)	0.0004	2.745 (0.992–7.598)	0.0519
Hypoalbuminemia: ≥2 vs. 0–1	3.452 (2.029–5.876)	<0.0001	2.570 (1.117–5.913)	0.0264	2.882 (1.647–5.042)	0.0002	1.309 (0.567–3.021)	0.5279
Creatinine: None vs. ≥1	1.304 (0.742–2.291)	0.3563	1.433 (0.748–2.744)	0.2778	1.480 (0.790–2.772)	0.2208	2.158 (0.980–4.752)	0.0563
LDH: ≥460 vs. <460	2.195 (0.691–6.979)	0.1826	2.751 (0.807–9.384)	0.1059	1.868 (0.591–5.903)	0.2874	3.682 (1.013–13.39)	0.0478
CRP: ≥3 vs. <3	2.547 (1.661–3.905)	<0.0001	1.612 (0.902–2.881)	0.1068	2.525 (1.620–3.935)	<0.0001	2.038 (1.141–3.642)	0.0161
Single agent chemotherapy vs. platinum doublet combination therapy	0.834 (0.588–1.183)	0.3098	0.836 (0.572–1.222)	0.3560	0.684 (0.470–0.995)	0.0468	0.629 (0.423–0.934)	0.0217

Abbreviations: BMI, body mass index; CCI, Charlson Comorbidity Index; CI, confidence interval; CRP, C‐reactive protein; ECOG‐PS, Eastern Cooperative Oncology Group performance status; HR, hazard ratio; LDH, lactate dehydrogenase; MMSE, Mini‐Mental State Examination.

We apologize for these errors.

