# Supplementary material for: Clinical benefit of platinum doublet combination therapy in older adults with advanced non‐small cell lung cancer: A prospective multicenter study by the National Hospital Organization in Japan
Source: Thorac Cancer. 2023 Apr 18;14(17):1597–605. doi: 10.1111/1759-7714.14904 (PMC10260489; doi:10.1111/1759-7714.14904)
Supplement: Supplementary file 1 — Table S1. Detail of regimen [file TCA-14-1597-s001.docx]

Supplementary Table 1. Detail of regimen

|  | Regimen | N |
| --- | --- | --- |
| Monotherapy | DTX | 26 |
|  | DTX + BEV | 3 |
|  | PEM | 16 |
|  | PEM + BEV | 4 |
|  | nab-PTX | 3 |
|  | S-1 | 3 |
|  | GEM | 1 |
|  | VNR | 1 |
|  | UFT | 1 |
| Combination | CBDCA + PEM | 32 |
|  | CBDCA + PEM + BEV | 10 |
|  | CBDCA + nab-PTX | 18 |
|  | CBDCA + nab-PTX + BEV | 1 |
|  | CBDCA + PTX | 7 |
|  | CBDCA + PTX + BEV | 8 |
|  | CBDCA + S-1 | 9 |
|  | CBDCA + GEM | 1 |
|  | CBDCA + VP-16 | 3 |
|  | CDDP + VP-16 | 1 |

DTX = Docetaxel; BEV = Bevacizumab; PEM = Pemetrexed; nab-PTX = nanoparticle albumin–bound paclitaxel; S-1 = Tegafur/Gimeracil/Oteracil; GEM = Gemcitabine; VNR = Vinorelbine; UFT = Tegafur/Uracil; CBDCA = Carboplatin; PTX = paclitaxel; VP-16 = Etoposide
